# Aging Reduces L-Type Calcium Channel Current and the Vasodilatory Response of Small Mesenteric Arteries to Calcium Channel Blockers

**DOI:** 10.3389/fphys.2016.00171

**Published:** 2016-05-20

**Authors:** Sulayma A. Albarwani, Fathi Mansour, Abdul Aleem Khan, Intisar Al-Lawati, Abdulla Al-Kaabi, Al-Manar Al-Busaidi, Safa Al-Hadhrami, Isehaq Al-Husseini, Sultan Al-Siyabi, Musbah O. Tanira

**Affiliations:** ^1^Department of Physiology, College of Medicine and Health Sciences, Sultan Qaboos UniversityMuscat, Oman; ^2^Department of Pharmacology and Clinical Pharmacy, College of Medicine and Health Sciences, Sultan Qaboos UniversityMuscat, Oman

**Keywords:** aging, voltage-gated calcium channel, calcium channel blockers, mesenteric arteries, F344 rats

## Abstract

Calcium channel blockers (CCBs) are widely used to treat cardiovascular disease (CVD) including hypertension. As aging is an independent risk factor for CVD, the use of CCBs increases with increasing age. Hence, this study was designed to evaluate the effect of aging on the sensitivity of small mesenteric arteries to L-type voltage-gated calcium channel (LTCC) blockers and also to investigate whether there was a concomitant change in calcium current density. Third order mesenteric arteries from male F344 rats, aged 2.5–3 months (young) and 22–26 months (old) were mounted on wire myograph to measure the tension during isometric contraction. Arteries were contracted with 100 mM KCl and were then relaxed in a cumulative concentration-response dependent manner with nifedipine (0.1 nM–1 μM), verapamil (0.1 nM–10 μM), or diltiazem (0.1 nM–10 μM). Relaxation-concentration response curves produced by cumulative concentrations of three different CCBs in arteries of old rats were shifted to the right with statistically significant IC50s. pIC_50_ ± s.e.m: (8.37 ± 0.06 vs. 8.04 ± 0.05, 7.40 ± 0.07 vs. 6.81 ± 0.04, and 6.58 ± 0.07 vs. 6.34 ± 0.06) in young vs. old. It was observed that the maximal contractions induced by phenylephrine and reversed by sodium nitroprusside were not different between young and old groups. However, Bay K 8644 (1 μM) increased resting tension by 23 ± 4.8% in young arteries and 4.7 ± 1.6% in old arteries. LTCC current density were also significantly lower in old arteries (−2.77 ± 0.45 pA/pF) compared to young arteries (−4.5 ± 0.40 pA/pF); with similar steady-state activation and inactivation curves. Parallel to this reduction, the expression of Ca_v_1.2 protein was reduced by 57 ± 5% in arteries from old rats compared to those from young rats. In conclusion, our results suggest that aging reduces the response of small mesenteric arteries to the vasodilatory effect of the CCBs and this may be due to, at least in part, reduced current density of LTCC.

## Introduction

One important functional change that is associated with aging and seen to occur in both humans and animals, is impaired vascular function (Ishida et al., 2003; Yildiz, 2007). The changes include alterations in endothelial function (Herrera et al., 2010), myogenic tone (Geary and Buchholz, 2003), vascular reactivity (Muller-Delp, 2006; Sinkler and Segal, 2014; Tümer et al., 2014), vascular calcium signaling (Georgeon-Chartier et al., 2013) and the expression of vascular ion channels (Albarwani et al., 2010; Fukuda et al., 2014). Calcium influx through dihydropyridine-sensitive L-type voltage-gated calcium channels (LTCC) plays a major role in the contractility of vascular smooth muscle cells (VSMCs; Gollasch and Nelson, 1997). In these resistance arteries, the influx of calcium ions through LTCCs regulates arterial tone and blood pressure (Moosmang et al., 2003).

LTCCs have high sensitivity to calcium channel blockers (CCBs) such as dihydropyridine, phenylalkylamine, and diltiazem (Triggle, 2007). Being potent vasodilator agents, these drugs are widely used as antihypertensive drugs. In resistance arteries, CCBs cause reduction in total peripheral resistance and hence lower blood pressure (Godfraind, 2014). CCBs bind to the α1 subunit of LTCC (α_1C_), though at different sites, and inhibit steady state calcium influx causing vasorelaxation (Lacinová, 2005). CCBs have been reported to cause more pronounced reduction in blood pressure in hypertensive humans (Leonetti et al., 1982) and in rats (Morel and Godfraind, 1994) than in their respective normotensives.

Several studies have reported an increased expression of LTCCs in hypertension in various arterial trees such as mesenteric, skeletal (Cox and Lozinskaya, 1995; Pratt et al., 2002), and renal arteries (Pesic et al., 2004), indicating the up-regulation of these channels in this disease, which is of high prevalence among the older population. However, no study has documented the influence of aging on vascular LTCCs and their sensitivity to CBBs. Del Corsso et al. (2006) reported that advanced age alters neither the number nor the activity of LTCCs in murine mesenteric artery. However, a recent study (Fukuda et al., 2014) reported attenuation of expression of LTCCs in the aorta (conduit artery) of old normotensive and spontaneously hypertensive rats. The study also showed that sensitivity of the LTCCs to nifedipine and verapamil was reduced in the aorta.

Based on these findings, we hypothesized that aging might be associated with a reduced expression of LTCCs in resistance mesenteric arteries, and this may subsequently attenuate the vasodilatory effect of CCBs. Therefore, the present study was designed to compare the LTCC current and the level of expression of their pore forming protein of VSMCs, isolated from resistance arteries of young and old rats. We also assessed their sensitivity to three different CCBs; diyhdropyridine (nifedipine), phenylalkylamine (verapamil), and benzothiazepine (diltiazem).

## Materials and methods

All procedures were performed after approval of Sultan Qaboos University (SQU) Animal Ethics Committee and in accordance to SQU Guidelines for Care and Use of Laboratory Animals.

### Animals

Male F344 rats, aging 2.5–3 months (young) and 22–26 months (old) were housed in Sultan Qaboos University Small Animal House Facility in a temperature-controlled room (22 ± 2°C) with a 12/12 h light/dark cycle and received food and water *ad libitum*. Rats were sacrificed with an overdose of a mixture of ketamine (140 mg kg^−1^ i.m.) and xylazine (40 mg kg^−1^ i.m.). Third order mesenteric arteries were isolate and placed in cold PSS solution of the following composition (mM): 119 NaCl, 4.7 KCl, 1.18 KH_2_PO_4_, 1.17 MgSO_4_, 25 NaHCO_3_, 5.5 glucose, and 1.6 CaCl_2_, pH 7.4 adjusted with NaOH.

To ensure that aging was not associated with changes in blood pressure, the mean blood pressure was measured in a sample of young and old rats (6 each). Animals were anesthetized using pentobarbetal (60 mg/Kg) of body weight. A polyethylene catheter with heparinized saline solution connected to an intravascular blood pressure transducer (iWorx/BP-102) was inserted into the right carotid artery. The pressure transducer was interfaced to a data acquisition system (iWorx/ETH-401), to continuously monitor the mean arterial blood pressure by a personal computer. Data were analyzed using (LabScribe3, iWorx) analysis software.

### Electrophysiological measurements

VSMCs were freshly dissociated from second and third branches of mesenteric arteries using a method previously described (Albarwani et al., 2010). Whole-cell records were made at room temperature using Axon 200B Amplifier. Inward currents were elicited with a progressive 8 mV pulse from a holding potential of −70 mV to +58 mV in a bath solution containing in (mM) 10 BaCl_2_, 135 TEA, 1 MgCl_2_, 10 HEPES, and 10 glucose. Pipette solution contained (mM) 145 Cs glutamate, 1 MgCl, 10 HEPES, 10 EGTA, and 1 Na_2_ATP at pH 7.3 titrated with CsOH. Current-voltage curves were plotted after dividing peak current to cell capacitance to normalize for cell size.

For voltage-dependent activation, current-voltage curves were used to calculate I/I_*max*_. For steady-state inactivation, a two-pulse voltage protocol was used; from −70 mV to a series of test potentials of 8 mV increments for 15 ms duration to +58 mV followed by depolarizing pulse to +10 mV for 500 ms. Voltage-dependent activation and inactivation curves were fitted by the Boltzmann equation: I/I_*max*_ = A_2_ + (A_1_ − A_2_)/(1 + exp (V − V_1∕2_)/s), where V is the command voltage, V_1∕2_ is the half-maximal potential, s is the slope factor, A_1_ is the highest value of the ratio I/I_*max*_ and A_2_ is the lowest value of the ratio I/I_*max*_ or G/G_*max*_. Data acquisition and analysis were performed using pClamp 9.2 (Molecular Devices, CA USA).

### Western immunoblotting

Western immunoblotting was performed as described by Albarwani et al. (2010). Equal amounts of mesenteric artery proteins (30 μg) were loaded into adjacent lanes. Membranes were incubated with monoclonal anti-Ca_v_1.2 calcium channel (1:300, NeuroMab, UCDavis) and with monoclonal β-actin antibody (dilution 1:1000, Santa Cruz, Biotechnology), both overnight at 4°C. After washing, membranes were incubated for 1 h at room temperature with the horseradish peroxidase-conjugated secondary antibodies (dilution 1:10,000, Santa Cruz, Biotechnology).

Immunoreactive bands corresponding to the molecular weight were detected by Supersignal West Dura Substrate (Thermo Scientific, life technology). Each protein sample was prepared from mesenteric arteries that were pooled from three rats. A total of four different samples were run for each animal group. Proteins were quantified using imageJ software normalized for loading differences to β-actin signal and expressed relative to young density.

### Simultaneous arterial diameter and intracellular calcium measurements

Arteries were incubated with 0.04% pluronic acid in PSS for 30 min then 2 μM Fura-2AM was added according to method described by Calderón-Sánchez et al. (2009). Arteries were then washed using PSS and mounted on a pressure myograph (DMT, Denmark), pressurized under no flow at 80 mmHg and incubated for an additional half an hour at 37°C. Diameters were recorded simultaneously with light emitted by arteries at 510 nm at excitation wavelength of 340 and 380 nm (Ionoptix Coorporation, MA, USA). Intracellular relative Ca^2+^ level was measured as the ratio of florescence intensities at R_340∕380_.

Arteries were first contracted with 60 mM KCl solution and then subsequently relaxed with nifedipine (1 μM). Differences in fura-2 signals (R_340∕380_) in the presence and absence of 60 mM KCl were considered as 100%. Changes in response to nifedipine were calculated as percentage of KCl response as follows:

$$\begin{array}{l} \text{Relaxation}(\%)=({\text{Diameter}}_{\text{KCl}}-{\text{Diameter}}_{\text{nifedipine}}/\\ \;{\text{Diameter}}_{\text{KCl}}-{\text{Diameter}}_{\text{baseline}})\times 100\\ \text{Calciumlevel}(\%)=({\text{Ratio}}_{\text{nifedipine}}-{\text{Ratio}}_{\text{baseline}}/{\text{Ratio}}_{\text{KCl}}\\ \;-{\text{Ratio}}_{\text{baseline}})\times 100 \end{array}$$

Where Diameter_KCl_ and Ratio_KCl_ are diameter and calcium ratio in the presence of 60 mM KCl. Diameter_nifedipine_ and Ratio_nifedipine_ are diameter and calcium ratio in the presence of nifedipine respectively and Diameter_baseline_ and Ratio_baseline_ are diameter and calcium ratio before contracting with KCl.

### Isometric tension recording

To obtain concentration response curves of CCBs, arteries were mounted on a wire myograph (DMT, Denmark), super fused with warm (37°C) PSS solution and tension was measured isometrically. Equal lengths of arterial segments were stretched progressively to an internal circumference equivalent to 90% of the circumference that vessels would have reached if exposed to 100 mmHg transmural pressure (Halpern et al., 1978). After the normalization procedure, arteries were left to equilibrate for 30 min at 37°C before subsequent evaluation. In all experiments, the viability of arteries was assessed at the beginning of experiment by a contractile response to phenylephrine (PE, 4 μM) and the integrity of the endothelium was assessed by a dilator response to 1 μM acetylcholine. Only arteries that showed relaxation of ≥80% for young and 60% for old were included in the study.

Arteries were contracted with 100 mM KCl to maximally depolarize the membranes then were relaxed with nifedipine (0.1 nM–1 μM), verapamil (0.1 nM–10 μM), or diltiazem (0.1 nM–10 μM). Concentration of each CCB was used twice and the average of two relaxations for each CCB concentration was calculated and reported. Differences in tensions between KCl-contraction and basal tension were considered as maximal tension (100%); relaxations to CCBs were expressed as the percentage of relaxation from the maximal response induced by 100 mM KCl.

To confirm that the relaxations in response to CCBs were not contributed to by non-specific ability of arteries from old rats to relax, a subset of arteries were contracted with PE (4 μM) and relaxed with a cumulative concentrations of a nitric oxide (NO) donor, sodium nitroprusside (SNP) (0.1 nM–10 μM).

The availability of LTCCs in causing arterial contractions was assessed by contracting the arteries with cumulative concentrations of the LTCC agonist, Bay K 8644 (0.1 nM–1 μM). Each vessel was used for only one CCB or for SNP.

### Chemicals

Nifedipine, PE, SNP, and acetylcholine (ACh) were obtained from Sigma (Germany). Diltiazem, Bay K 8644, verapamil from Tocris (UK), and fura-2 AM was obtained from Molecular Probes (USA).

### Data analysis

The concentrations of CCBs that produced half maximal responses (IC_50_) were calculated using non-linear regression analysis (GraphPad Prism Software, San Diego, CA, USA). The IC_50_ values were expressed as negative logarithm of molar concentration (pIC_50_). Electrophysiology data were analyzed using Clampfit (Molecular Devices, Sillicon Valley USA) and Microcal Origin (Northampton MA). Values are mean ± s.e.m. Statistical comparisons between groups were made with one-way, ANOVA with subsequent Tukey *post-hoc* analysis test. *p* level of ≤ 0.05 was considered significant.

## Results

### Blood pressure

Weight of young rats were 299.08 ± 6.11 gm and of old rats were 385.41 ± 6.8 gm. Blood pressure (systolic/diastolic) of young rats was 111.1 ± 7.1/91.9 ± 7.9 and the old rats were 101.8 ± 4.5/78.8 ± 3.2. The low blood pressure of old rats was not significantly different from that of young rats (*n* = 6 each).

### LTCC currents

**Figure 1:**
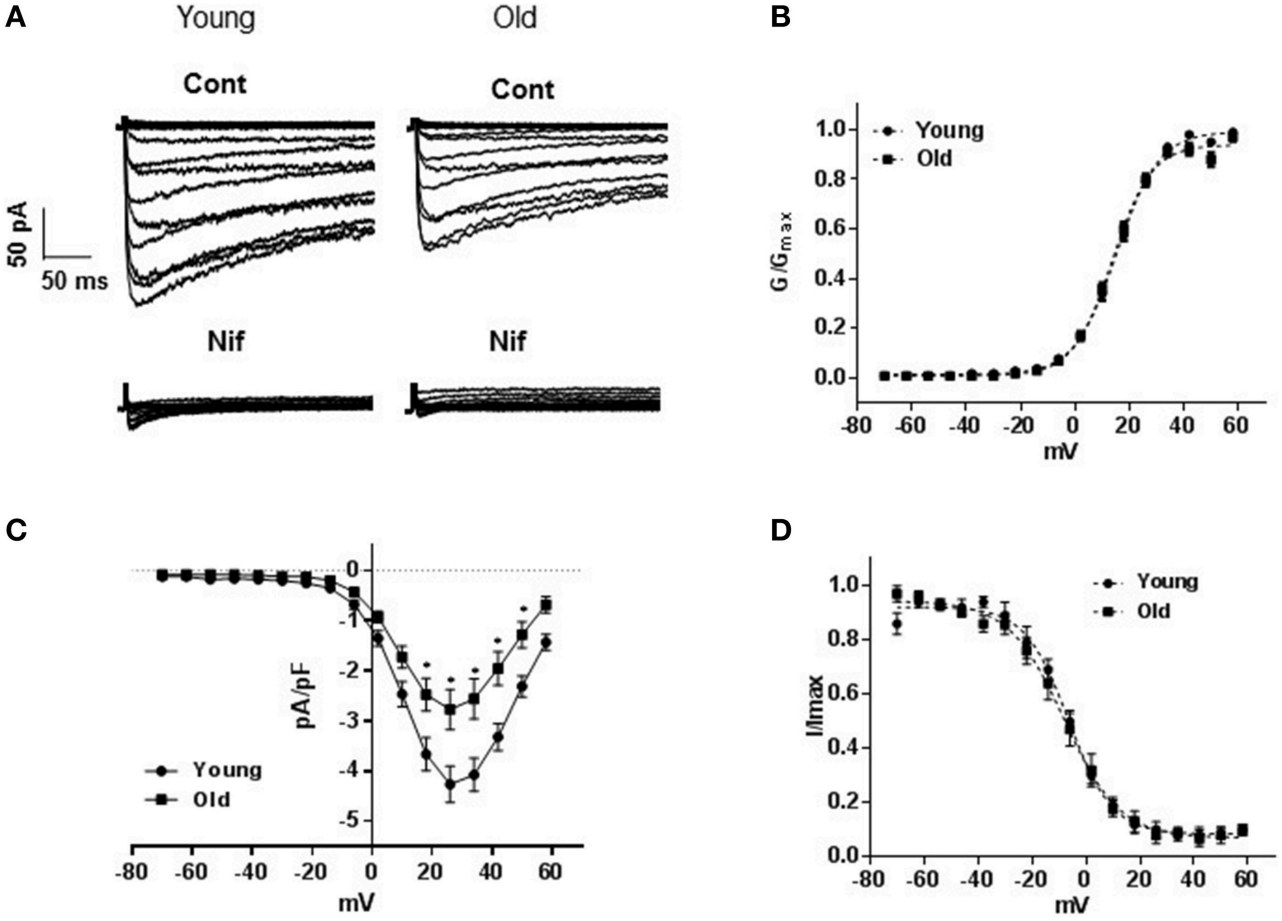
**L-type voltage-gated calcium channel currents**. **(A)** Representative traces of inward Ba^2+^ currents generated by 8 mV steps from a holding potential of −70 to +58 mV in vascular smooth muscle cells (VSMCs) isolated from mesenteric arteries of young and old rats. Membrane capacitances were 32 and 48 pF, respectively. Currents from both animal groups (cont) were equally blocked by 1 μM nifedipine (nif). **(B)** Averaged current-voltage (I-V) relationships showing current density in VSMCs of arteries from young compared to old. Steady state activation **(C)**, and inactivation **(D)** curves are represented as I/I_max_ and G/G_max_; respectively.

Inward currents recorded from cells isolated from old rats were significantly lower than those obtained from cells isolated from young animals. Nifedipine (1 μM) reduced the inward currents by approximately 90% in both groups indicating that the inward currents were mainly of LTCC type (Figure 1A). Peak current densities recorded at +26 mV were −4.5 ± 0.40 pA/pF (young, *n* = 17 cells) and −2.77 ± 0.45 pA/pF (old, *n* = 17 cells; *P* = 0.001, Figure 1B). Analysis of activation (Figure 1C) and inactivation curves (Figure 1D) revealed statistically insignificant differences in half-maximal voltages (V_0.5_) and slopes. Activation V_0.5_ were 13.7 ± 0.7 mV (*n* = 17 cells) and 15.0 ± 0.67 mV (*n* = 16 cells) and inactivation V_0.5_ were −8.02 ± 0.7 mV (*n* = 5 cells) and −6.34 ± 0.67 mV (*n* = 7 cells) for arteries from old and young rats; respectively.

### Expression level of Ca_v_1.2

**Figure 2:**
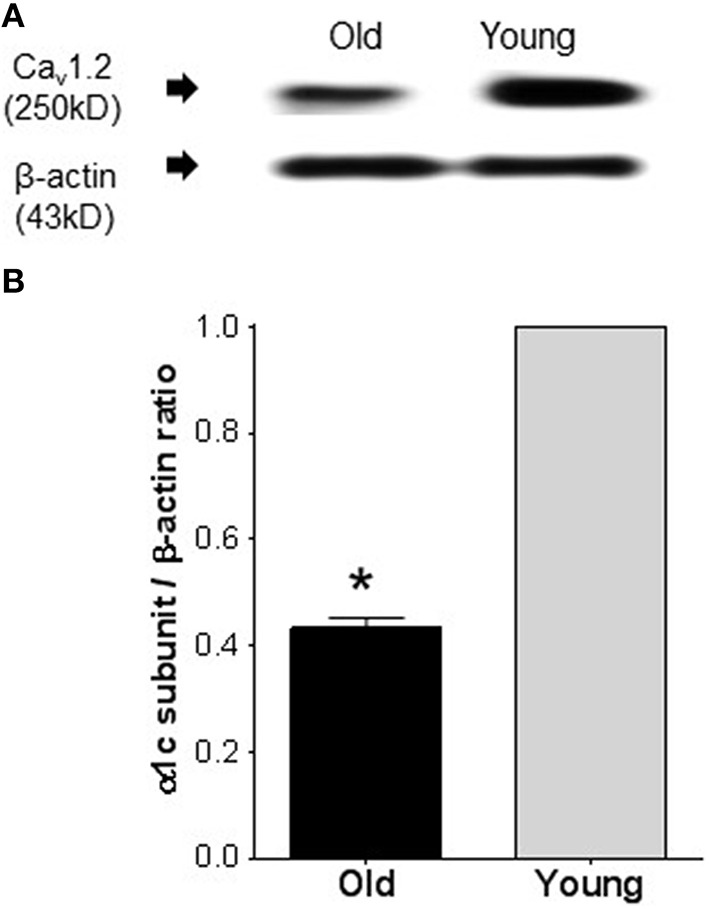
**Western immunoblots comparing the expression of Ca_**v**_1.2 channel protein in arteries from old and young rats**. **(A)** Representative band densities of Ca_v_1.2 channel protein isolated from mesenteric arteries of old and young rats. Band densities for the internal standard, β-actin, of the same blot is shown in the lower panel. **(B)** Average densitometric values of normalized Ca_v_1.2 to β-actin densities from four Western blots, indicating reduced Ca_v_1.2 channel protein expression in mesenteric arteries of old compared to young rats. Bars represent means ± S.E.M. ^*^Significantly different from young (*P* < 0.0001).

Figure 2 shows a representative blot density of Ca_v_1.2 protein isolated from old and young resistance arteries. Western blot revealed a significant (*P* ≤ 0.0001) reduction of the pore forming Ca_v_1.2 protein in old compared to that the young arteries. Samples from old arteries showed 57 ± 5% reduction in the protein density.

### Contractile response of arteries induced by LTCC opener

**Figure 3:**
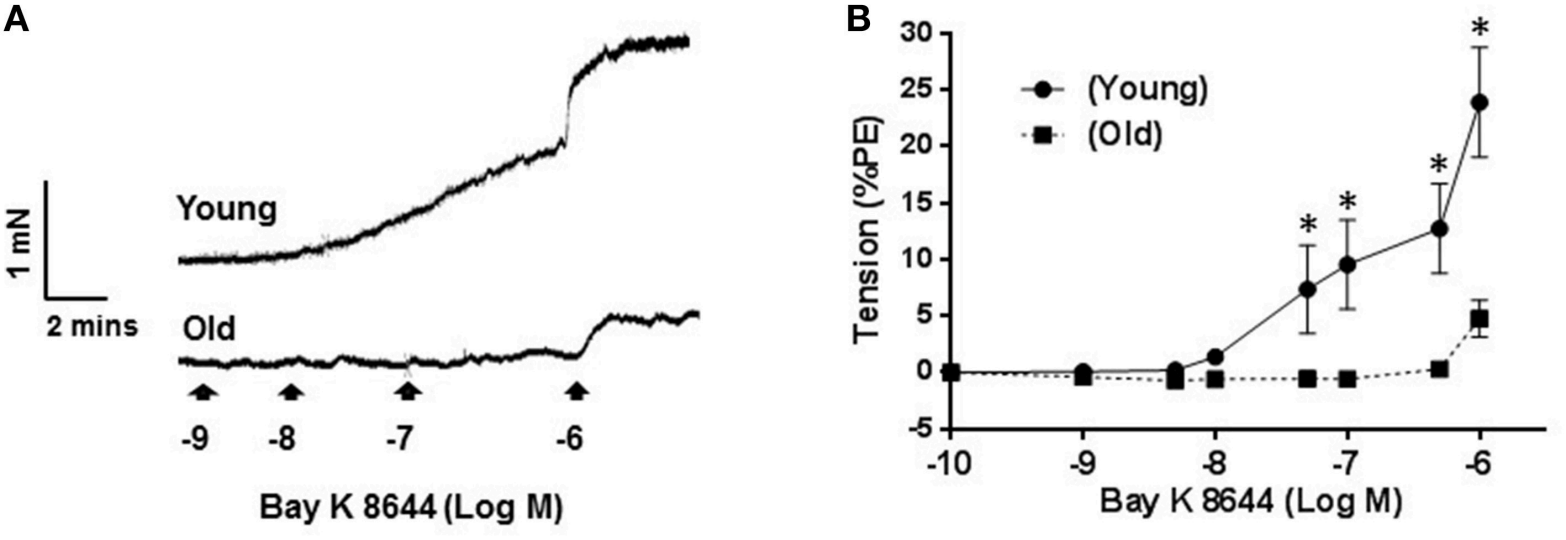
**Effect of Bay K 8644 on basal tension**. Representative tension recording traces of segments of mesenteric arteries isolated from young and old rats that were treated with cumulative concentrations of L-type calcium channel agonist, Bay K 8644 **(A)**. Average concentration-response curves of Bay K 8644 induced-contraction plotted as percentage of phenylephrine (PE) contraction **(B)** for arteries from young and old rats. Values are mean ± SEM, ^*^denotes significance at *P* ≤ 0.05.

Figure 3A shows representative traces of isometric tension recordings, demonstrating the effect of Bay K 8644 on resting tension of mesenteric arteries. Bay K 8644 produced concentration-dependent increases in tensions, which were more pronounced in arteries isolated from young than old rats. At the maximum concentration of Bay K 8644 used (1 μM), arteries from young rats contracted by 23 ± 4.8% of KCl-contraction compared to 4.7 ± 1.6% in arteries isolated from old rats (*P* ≤ 0.0001; Figure 3B, *n* = 5).

### Relaxation response of arteries induced by LTCC blockers

Contractions induced by 100 mM KCl were 10.8 ± 0.6 vs. 10.7 ± 0.7 mN and those elicited by PE (4 μM) were 13 ± 0.5 vs. 12 ± 0.8 mN; young (*n* = 27) vs. old (*n* = 28); respectively. Both contractile responses were not significantly different between the two groups. Relaxation in response to 1 μM ACh were 91.07 ± 3.99% and 80.01 ± 4.3% of PE-induced contraction for arteries from young and old rats, respectively (*p* ≤ 0.01).

**Figure 4:**
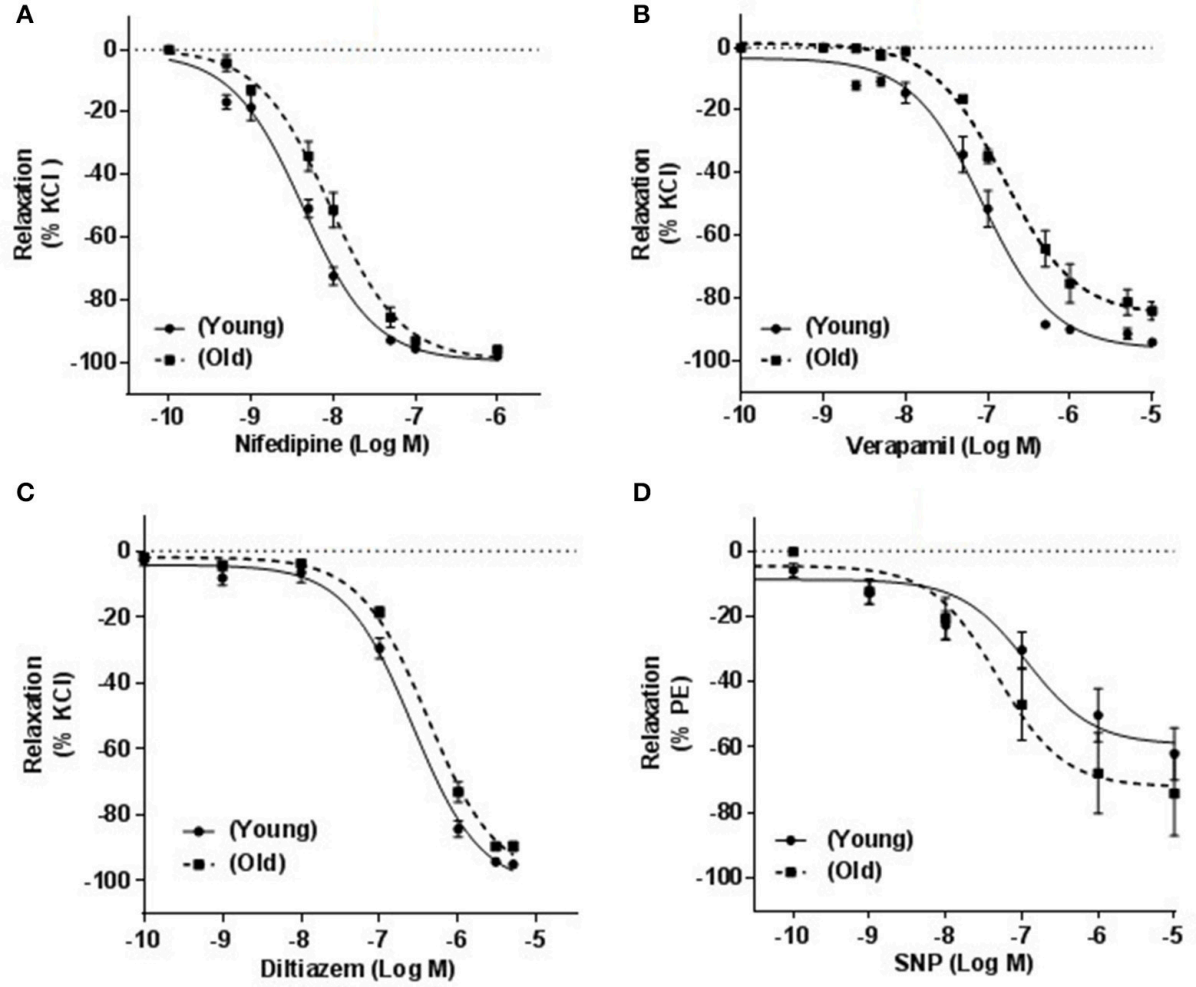
**Effect of calcium channel blockers on KCl-induced contraction**. Responses of mesenteric arteries isolated from young and old rats that were pre-contracted with 100 mM KCl and relaxed with cumulative concentrations of three calcium channel blockers, nifedipine **(A)**, verapamil **(B)**, and diltiazem **(C)**. **(D)** Shows the relationship between cumulative concentrations of sodium nitroprusside (SNP) and % relaxation of phenylephrine (PE; 4 μM)-induced contraction.

Relaxation-response curves produced by cumulative concentrations of three different CCBs on arteries of old rats were shifted to the right with statistically significant IC_50_s. pIC_50_ ± s.e.m were: 8.37 ± 0.06 vs. 8.04 ± 0.05 (*p* = 0.0004) for nifedipine (*n* = 8; Figure 4A), 7.40 ± 0.07 vs. 6.81 ± 0.04 (*p* = 0.0061) for verapamil (*n* = 7; Figure 4B) and 6.58 ± 0.07 vs. 6.34 ± 0.06 (*p* = 0.034) for diltiazem (*n* = 12, Figure 4C) in young vs. old. The maximum relaxations (%) for nifedipine were 98.00 ± 0.10 and 96.00 ± 0.20 (*p* ≤ 0.01), for verapamil were 91.32 ± 1.12 and 84.80 ± 2.35 (*P* ≤ 0.001) and for diltiazem were 94.98 ± 1.01 and 89.51.98 ± 1.40 (*p* ≤ 0.001) for arteries from young and old rats, respectively.

To investigate whether these differences in vascular relaxations were specifically associated with CCBs, arteries were relaxed with SNP in a cumulative concentration response manner (0.1 nM to 10 μM). Maximum relaxations were 62.02 ± 7.96 and 74.2 ± 12.92 (*p* = 0.2193) and pIC_50_ were 6.93 ± 0.32 and 7.32 ± 0.17 (*p* = 0.1424) in arteries obtained from young compared to old rats (*n* = 8, Figure 4D).

### Changes in diameters of arteries and intracellular calcium in response to nifedipine

**Figure 5:**
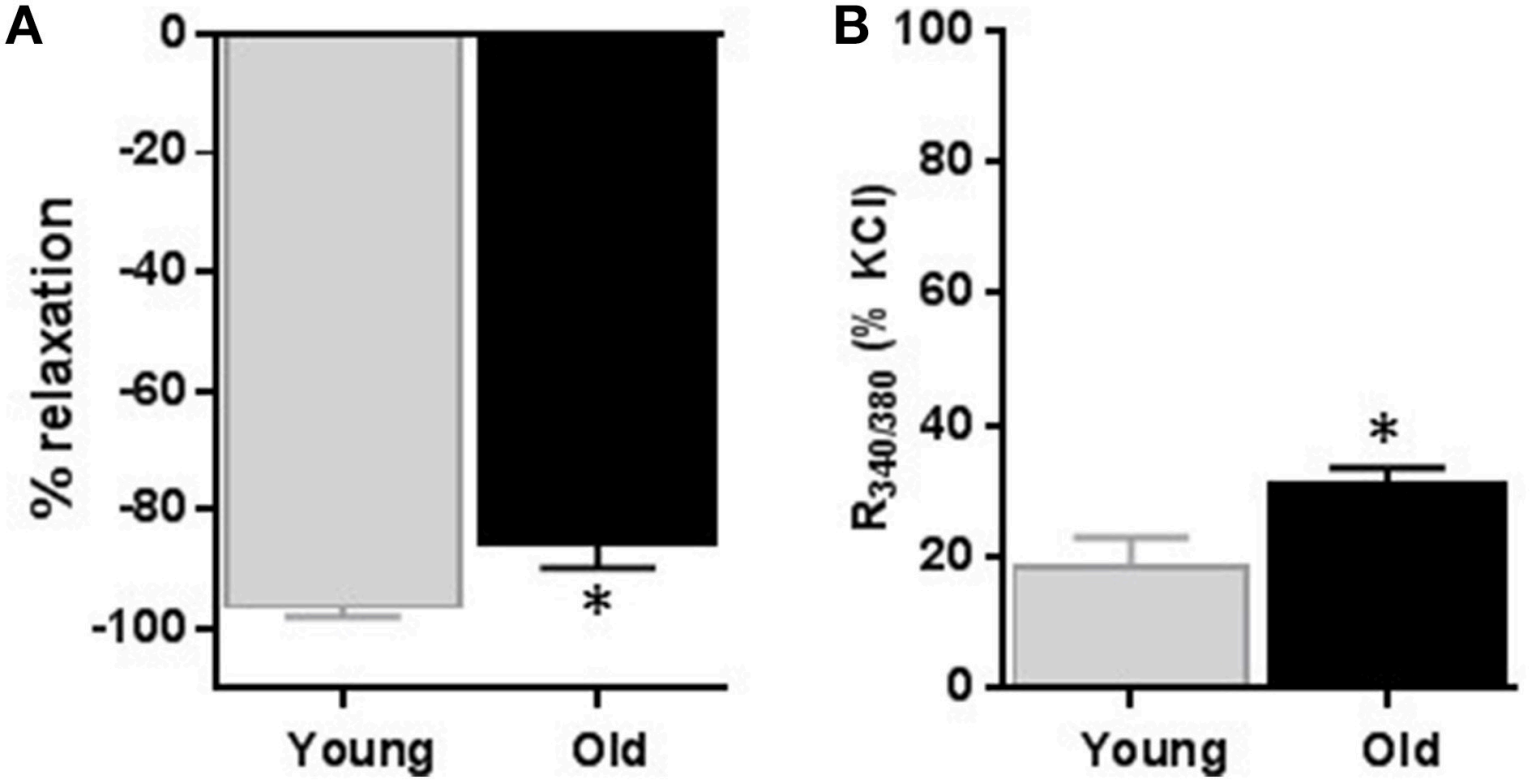
**Effect of nifedipine on diameter and relative intracellular calcium level changes**. Average data of diameter changes **(A)** and intracellular calcium level as indicated by R340/380 **(B)** of small mesenteric arteries isolated from young and old rats that were pre-contracted with 60 mM KCl and then relaxed with 1 μM nifedipine. Values are mean ± SEM of % changes from KCl contraction. ^*^denotes significance at *P* ≤ 0.05.

To observe changes in the steady state influx of calcium associated with the relaxation caused by CCBs, we simultaneously recorded changes in arterial diameter and Fura-2 signal after nifedipine (1 μM). Nifedipine relaxed KCl-induced contraction by 96.2 ± 1.6% vs. 85.5 ± 4.3% (*p* = 0.023, Figure 5A, *n* = 6); whereas it reduced the R_340∕380_ signal to 18.6 ± 4.3% vs. 30.6 ± 3.0% in arteries obtained from young vs. old rats; respectively (*p* = 0.0354, Figure 5B, *n* = 6).

## Discussion

The present study endeavored to understand the role of LTCCs in age-related alterations in mesenteric arterial smooth muscle cells (MASMCs) responsiveness. It is well-known that the main pathway for calcium entry in these excitable cells is via LTCCs (Catterall, 2011), hence, any change in LTCCs, we hypothesized, may alter the function of VSMCs and their response to their agonists and antagonists.

Electrophysiological experiments were conducted to compare the density of LTCC type current, its voltage dependence and blockade by the CCB (nifedipine), as nifedipine was shown to be the most potent blocker of the VSMC channels compared to verapamil and diltiazem (Morel et al., 1998; Godfraind, 2014). Our results demonstrated an age-dependent reduction in LTCC current density in the isolated MASMCs, of 22–26 months (old) compared to 2.5–3 months (young) male F344 rats. In our experiment, the inward currents recorded from small arteries of both young and old animals were almost completely abolished by 1 μM nifedipine, despite the fact that the VSMCs isolated from old rats showed statistically significant reduction in current density. Although we did not record single channel activity, the similarities in whole-cell steady-state activation and inactivation curves may indicate that the reason for the observed reduced current density is most likely to be due to reduced current density rather than its voltage dependence.

Lozinskaya and Cox (1997) studied the effect of aging on LTCC currents in small mesenteric artery myocytes from Wistar-Kyoto rats (WKY) and SHR juvenile (5–7 weeks), young (10–12 weeks) and mature (19–23 weeks) and did not find any significant differences in LTCC current density of normotensive rats with progress of age. This difference could possibly be due to age difference, as in their study 26 months rats were not used or/and due to difference of strains. Probably this would require some more experimental work to clarify this incongruity.

The age-dependent reduction in LTCC current density in the isolated MASMCs was further corroborated by the significant reduction in expression of Ca_v_1.2 protein that forms the pore of LTCCs. The reduced expression of Ca_v_1.2 protein substantially lowered the sensitivity of small mesenteric arteries to CCBs. Our results are further supported by a recent study (Fukuda et al., 2014), where they demonstrated reduced LTCC protein expression an effect that was associated with reduced CCBs-induced relaxation in aorta of 40 weeks old rats. However, it will not be appropriate to extrapolate the results obtained from the large conduit arteries to small resistance arteries as it has been shown conduit and resistance arteries have different response properties to CCBs (Leloup et al., 2015). Our study provided a direct electrophysiological evidence to suggest that an age dependent reduction in LTCC current and the associated reduced current density could more likely be due to lowered expression of LTCC as indicated by reduced expression of the pore forming Ca_v_1.2 protein.

In order to evaluate the effect of aging on the sensitivity of small mesenteric arteries to CCBs, we further investigated the effect of CCBs on KCl-precontracted mesenteric arteries isolated from old and young rats. In VSMCs, high extracellular K^+^ depolarizes cell membranes causing opening of LTCCs which allow Ca^2+^ influx and, in turn, causes contraction of arterial smooth muscle (Harder et al., 1983). Our results demonstrated that KCl-induced contractions were effectively reversed by nifedipine, verapamil, or diltiazem. However, old rats' arteries were less sensitive to CCBs compared to arteries of young rats as indicated by the rightward shift of concentration response curves with statistically significant IC_50_. The three CCBs were shown to bind to different sites on the pore forming C_1α_ subunit of the LTCC (Opie, 1997; Abernethy and Schwartz, 1999). Thus, it may be possible that the lesser sensitivity of arteries shown to different CCBs in old rats could likely be related to LTCC itself rather than a specific CCB.

As age-related changes in sensitivity to different CCBs have been reported earlier in the aorta (Karaki et al., 1985; Fukuda et al., 2014) as well as in the pulmonary artery and vein (Ricci et al., 2000). Our study further showed that this age-associated reduction in vasodilation in response to CCBs also occurs in small arteries that contribute to peripheral resistance and in turn to increased blood pressure. To confirm that the observed rightward shift was not caused by a non-specific impaired ability of VSMCs of old rats' arteries, we contracted the arteries with phenylephrine and used a NO donor (SNP) to elicit LTCC-independent relaxation. The fact that relaxations induced by SNP were not significantly different in arteries from both groups, may imply that the observed rightward shift in response to CCBs was particularly associated with calcium channel blocking rather than due to an impaired relaxation of non-specific nature due to aging.

To determine the functional implications of reduced LTCC current, we increased calcium influx through LTCC current without depolarizing the membrane and monitored changes in resting tensions. Bay K 8644 has been reported to increase mean open time of LTCC to near 1, hence it has been argued that this increases basal tensions of arteries that is dependent on Ca^2+^ influx through LTCC (Nakayama and Brading, 1996). Therefore, Bay K 8644-induced contractions are supposed to be caused by an influx of Ca^2+^ through prolonged opened LTTCs (Nakayama and Brading, 1996). In studies where density of LTCC were shown to be increased, such as in spontaneously hypertensive rats compared to WKY arteries, Bay K 8644 has also been consistently shown to cause a larger increase in resting tone (Hernandez et al., 1995; Matsuda et al., 1997; Fukuda et al., 2014). Consistent with these findings, our results showed that contractile responses to Bay K 8644 were attenuated in old arteries compared to young ones, which is in support of the notion that in small mesenteric arteries of old rats there is reduced availability of functional LTCC. But, the discrepancy between the large magnitude of KCl—induced contractions and the abolished response to Bay K 8644 in arteries from old rats is not clear since both agents rely on Ca^2+^ influx through LTCC. One possible explanation would be what has been reported earlier that KCl may also increases sensitivity of contractile filaments to Ca^2+^ (Ratz et al., 2005), and hence, the tension observed in response to KCl could also be due to enhanced in Ca^2+^ sensitivity in addition to increases in Ca^2+^ influx. In this respect it is worth noting that this “unusual” disparity between KCl and Bay K8644 effect in our work has also been noticed by Hirenallur-S et al. (2008) who showed pulmonary arteries neonatal piglets to contract to KCl whereas, under the same condition Bay K 8644 showed no effect. Furthermore, it was showed that aging reduced the sensitivity of contractile myofilament to Ca^2+^ despite preservation of contractile responses has been demonstrated in mesenteric arteries of aged rats (Matz et al., 2003).

Finally, we measured relative intracellular calcium level during relaxation in response to nifedipine. We simultaneously compared the degree of relaxation in response to nifedipine with the level of intracellular calcium in KCl pre-contracted arteries. In line with our isometric tension recording experiments where we found that old arteries relaxed less to nifedipine, we also found that ${\text{Ca}}_{}^{2+}$ relative level, at the end of nifedipine-induced relaxation, was significantly more in arteries obtained from old than those obtained from young rats. The later effect could possibly be due to reduced LTCC sensitivity to nifedipine or reduced uptake by SERCA (Matz et al., 2003). Alternatively, it could merely reflect differences in Ca^2+^ influx during depolarization between the arteries from the two groups. It should be noted that, in our experiments we measured relative changes in [Ca^2+^]_i_ level rather than absolute changes in [Ca^2+^]_i_ and hence it cannot be decisively concluded that the [Ca^2+^]_i_ was different between the two groups.

In conclusion, our results suggest that aging reduces LTCC current density as well as the vasodilatory response of mesenteric resistance arteries to CCBs. The fact that the three CCBs bind to different sites on LTCC subunit, coupled with the observed lower sensitivity to CCBs and in addition to unchanged SNP effect support our findings that aging causes reduced LTCCs current density by down regulating α_1C_ subunit of LTCC which could manifest in the reduction of relaxing response to CCBs.

## Author contributions

S. Albarwani, AK, and MT, idea and manuscript preparation. FM performed electrophysiological experiments. I. Al-Lawati performed western blotting and some tension myography experiments. A. Al-Kaabi, A. Al-Busaidi, S. Al-Hadhrami, and I. Al-Husseini, performed calcium channel blockers experiments, S. Al-Siyabi performed Fura experiments.

## Funding

This work was funded by The National Research Council grant # RC/SQU/MED/PHYS/12/01 to SA.

### Conflict of interest statement

The authors declare that the research was conducted in the absence of any commercial or financial relationships that could be construed as a potential conflict of interest.

## Abbreviations

Bay K 8644, 1, 4-Dihydro-2: 6-dimethyl-5-nitro-4-[2-(trifluoromethyl) phenyl]-3-pyridinecarboxylic acid methyl ester.
